# LncRNA NEAT1 promotes proliferation, migration, invasion and epithelial-mesenchymal transition process in TGF-β2-stimulated lens epithelial cells through regulating the miR-486-5p/SMAD4 axis

**DOI:** 10.1186/s12935-020-01619-8

**Published:** 2020-10-31

**Authors:** Huajun Wang, Guangying Zheng

**Affiliations:** Department of Ophthalmology, The First Affiliated Hospital of Zhengzhou University, No. 1 Jianshe East Road, Zhengzhou, 450000 Henan China

**Keywords:** Posterior capsular opacification, TGF-β2, NEAT1, miR-486-5p, SMAD4

## Abstract

**Background:**

Abnormal proliferation, metastasis and epithelial-mesenchymal transformation (EMT) of lens epithelial cells (LECs) are direct factors of posterior capsular opacification (PCO). Nuclear enriched abundant transcript 1 (NEAT1) has been shown to promote cell proliferation, metastasis and EMT, but whether it affects the progression of PCO is unclear.

**Methods:**

The expression of NEAT1, microRNA-486-5p (miR-486-5p) and *Drosophila* mothers against decapentaplegic 4 (SMAD4) was determined using quantitative real-time polymerase chain reaction (qRT-PCR). The proliferation of cells was measured via 3-(4, 5-dimethyl-2 thiazolyl)-2, 5-diphenyl-2-H-tetrazolium bromide (MTT) assay. Transwell assay was employed to detect the migration and invasion of cells. The levels of EMT marker proteins, SMAD4 protein and transforming growth factor-β (TGF-β)/SMAD signaling pathway-related proteins were assessed by western blot (WB) analysis. Further, the relationship between miR-486-5p and NEAT1 or SMAD4 was confirmed by dual-luciferase reporter assay, RNA immunoprecipitation (RIP) assay and biotin-labeled RNA pull-down assay.

**Results:**

NEAT1 is upregulated and miR-486-5p is downregulated in the posterior capsular tissues of PCO patients and TGF-β2-induced LECs. Interference of NEAT1 reverses the promoting effect of TGF-β2 on the proliferation, migration, invasion and EMT of LECs. MiR-486-5p can be sponged by NEAT1, and its inhibitor reverses the suppression effect of NEAT1 silencing on the progression of TGF-β2-induced LECs. SMAD4 functions as a target of miR-486-5p, and its overexpression recovers the inhibition effect of miR-486-5p overexpression on the progression of TGF-β2-induced LECs. The activity of the TGF-β/SMAD signaling pathway is regulated by the NEAT1/miR-486-5p/SMAD4 axis.

**Conclusion:**

Our study shows that NEAT1 has a positive effect on the progression of PCO and is expected to become a new target for PCO treatment.

## Highlights

1. Depletion of NEAT1 suppresses the biological functions of TGF-β2-stimulated LECs;
2. NEAT1 directly interacts with miR-486-5p;
3. MiR-486-5p targets SMAD4;
4. The NEAT1/miR-486-5p/SMAD4 axis regulates the activity of the TGF-β/SMAD signaling pathway.

## Background

After extracapsular cataract extraction or after ocular trauma crystal rupture, the remaining cortex and capsular membrane become cloudy are called after cataract, also known as secondary cataract [1]. Modern secondary cataract mainly refers to posterior capsular opacification (PCO) [2, 3]. Current studies suggest that the proliferation, migration and epithelial-mesenchymal transformation (EMT) of lens epithelial cells (LECs) are the key factors for PCO formation [4, 5]. The EMT process of LECs plays a central role in the formation of PCO, which can lead to cell adhesion and loss of the apical-basal polarity of the mesenchymal phenotype, leading to the production of fibroblasts [6, 7]. Although the cause of PCO has been clarified, the molecular targets that influence its occurrence still need to be further explored. At present, transforming growth factor-β2 (TGF-β2)-induced LECs are considered to be an effective way to construct an in vitro model of PCO [8, 9], which provides a convenient experimental model for us to carry out relevant research on PCO.

Long non-coding RNA (lncRNA) is a non-coding protein that participates in the regulation of various processes in cells [10]. Research has confirmed that lncRNA can regulate cell growth, differentiation and apoptosis, and is related to many diseases progression [11, 12]. More importantly, many lncRNAs have been shown to regulate LECs proliferation, migration and EMT, such as FEZF1-AS1, HOTAIR and MIAT [13–15]. Nuclear enriched abundant transcript 1 (NEAT1) is a nuclear-restricted lncRNA that is abnormally expressed in many diseases and is thought to be associated with disease progression. Xiong et al. report that NEAT1 promotes proliferation, migration and invasion of cells to enhance the progression of breast cancer [16]. And Wang et al. show that NEAT1 regulates the EMT process of diabetic nephropathy [17]. Therefore, NEAT1 may play a vital function in cell proliferation, migration and EMT process. In PCO, Dong et al. conducts microarray analysis on the LECs of PCO patients and normal humans and reveals that the expression of NEAT1 in PCO is significantly increased [18]. However, it is unclear whether NEAT1 participates in the regulation of PCO progress.

Studies on the functions of microRNAs (miRNAs) have been largely confirmed by many researchers. In the regulatory network of lncRNA-miRNA-messenger RNA (mRNA), the role of miRNA as a bridge between lncRNA and target genes has also become the key to elucidate the molecular mechanism of lncRNA [19, 20]. MiR-486-5p is involved in the mediation of proliferation, metastasis and EMT of many diseases, including breast cancer and hepatocellular carcinoma [21, 22]. The study confirms that miR-486-5p shows a low expression trend in TGF-β2-induced LECs and can participate in the proliferation, invasion and EMT of TGF-β2-induced LECs [23].

*Drosophila* mothers against decapentaplegic 4 (SMAD4) belongs to the SMAD family and is activated by transmembrane serine/threonine receptor kinases, such as TGF-β receptors [24]. SMAD family proteins play an essential role in the transduction of TGF-β signaling from cell surface receptors to the nucleus [25]. The effects of SMAD4 on LECs proliferation and EMT have been reported in many studies [18, 26]. Therefore, the study of SMAD4 and TGF-β/SMAD signaling pathway will help us better understand the factors affecting the progression of PCO. Our study is aimed to explore the role of NEAT1 in PCO progression and the potential mechanism, hoping to provide a novel molecular target for the exploration of the prevention and control of PCO.

## Materials and methods

### Sample tissues collection

This study was approved by the ethics committee of The First Affiliated Hospital of Zhengzhou University and was performed in accordance with the Declaration of Helsinki. Posterior capsular tissues were obtained from 30 PCO patients (30 eyes, age range was 50–75, free of other ocular diseases) in The First Affiliated Hospital of Zhengzhou University. All patients were diagnosed with PCO and were graded: 6 cases of grade I, 21 cases of grade II, and 3 cases of grade III. Similarly, we also obtained normal posterior capsular tissues from 30 organ donors (age range was 45–72). Written informed consent was signed from each patient and donor.

### Cell culture, TGF-β2 treatment and cell transfection

Human LECs (SRA01/04) were bought from Biovector (Beijing, China). The cells were cultured in Dulbecco’s modified Eagle’s medium (DMEM; 12,100-046, Invitrogen, Carlsbad, CA, USA) containing 10% fetal bovine serum (FBS; 10437028, Invitrogen) and 1% penicillin/streptomycin (15140148, Invitrogen). SRA01/04 cells were incubated at 37 °C with 5% CO_2_ incubator. When cells reached 60% confluence, SRA01/04 cells were treated with different concentrations of TGF-β2 (0, 1, 5, and 10 ng/mL) for 48 h, and the expression of NEAT1, miR-486-5p and SMAD4 could be detected. Similarly, when cells reached 50% confluence, transfection could be performed, followed by treatment with 10 ng/mL TGF-β2. All vectors and oligonucleotides were purchased from RiboBio (Guangzhou, China), including NEAT1 small interfering RNA and pcDNA overexpression vector (si-NEAT1: 5′-GAGCAATGACCCCGGTGACG-3′and NEAT1: F 5′-TTGGGACAGTFFACGTGTGG-3′, R 5′-TCAAGTCCAGCAGAGCA-3′) or their negative controls (si-NC: 5′-TAGATACCCCCAGGCCTACC-3′; and pcDNA: 5′-TAGAAGGCACAGTCGAGG-3′), miR-486-5p mimic and inhibitor (miR-486-5p: 5′-UCCUGUACUGAGCUGCCCCGAG-3′; and anti-miR-486-5p: 5′-CUCGGGGCAGCUCAGUACAGGA-3′) or their negative controls (miR-NC, 5′-UUCUCCGAACGUGUCACGUTT-3′; and anti-miR-NC: 5′-CAGUACUUUUGUGUAGUACAA-3′), SMAD4 overexpression vector (SMAD4: F 5′-CGGACATTACTGGCCTGTTC-3′, R 5′-TAGGGCAGCTTGAAGGAAACC-3′) and its negative control (pcDNA: 5′-TAGAAGGCACAGTCGAGG-3′). Lipofectamine 3000 (L3000015, Invitrogen) was employed to transfect all vectors and oligonucleotides into SRA01/04 cells.

### Quantitative real-time polymerase chain reaction (qRT-PCR)

TRIzol reagent (15596-026, Invitrogen) was used to extract total RNA, and BeyoRT II cDNA Synthesis Kit (D7170S, Beyotime, Shanghai, China) was employed to synthesize cDNA. The NEAT1 and SMAD4 expression was measured using SYBR Green (11762500, Invitrogen) and glyceraldehyde 3-phosphate dehydrogenase (GAPDH) was designated as an internal control. The miR-486-5p expression was determined by TaqMan MicroRNA Assay (4440887, Applied Biosystems, Foster City, CA, USA) and U6 was employed as an internal control. All primers were as follows: NEAT1: F 5′-TTGGGACAGTFFACGTGTGG-3′, R 5′-TCAAGTCCAGCAGAGCA-3′; SMAD4: F 5′-CGGACATTACTGGCCTGTTC-3′, R 5′-TAGGGCAGCTTGAAGGAAACC-3′; miR-486-5p: F 5′-CGCGTCCTGTACTGAGCTGCC-3′, R 5′-ATCCAGTGCAGGGTCCGAGG-3′; U6: F 5′-CTCGCTTCGGCAGCACA-3′, R 5′-AACGCTTCACGAATTTGCGT-3′; GAPDH: F 5′-ACAGTCAGCCGCATCTTCT-3′, R 5′-GACAAGCTTCCCGTTCTCAG-3′. Data were analyzed using the 2^−ΔΔCt^ method.

### Cell proliferation assay

This assay was employed using the 3-(4, 5-dimethyl-2 thiazolyl)-2, 5-diphenyl-2-H-tetrazolium bromide (MTT) Assay Kit (C0009, Beyotime). SRA01/04 cells were collected at 24 h after transfection and inoculated on 96-well plates. After cells were attached to the wall, 10 μL MTT solution was added to each well and incubated in the incubator for 4 h. After removed supernatant, Formazan solvent was added to each well and shock dissolved for 10 min. Cell absorbance was measured at 490 nm and cell viability was calculated. Non-treated and non-transfected cells were used as Control.

### Transwell assay

This assay was performed using transwell chambers (3422, Corning Inc., Corning, NY, USA), which were pre-coated with Matrigel (354234, Corning Inc.) to measure the number of invaded cells and non-coated to determine the number of migrated cells. After treatment or transfection for 24 h, SRA01/04 cells were seeded in the upper chambers, which were filled with serum-free DMEM. In the lower chambers, DMEM contained 10% FBS was added. After 24 h, the cells were fixed with 4% methanol, stained using 0.1% crystal violet. The cells were photographed (100×) and counted using a microscope (DM500, Leica, Wetzlar, Germany). Non-treated and non-transfected cells were used as Control.

### Western blot (WB) analysis

SRA01/04 cells were lysed using RIPA reagent (P0013K, Beyotime) containing protease inhibitor cocktail (Beyotime). After that, total protein was quantified using BCA Kit (Beyotime). 30 μg protein samples were subjected to 10% separating gel and transferred to polyvinylidene difluoride (PVDF) membranes (Millipore, Billerica, MA, USA). Next, the membranes were blocked with 5% nonfat milk, incubated with primary antibodies and probed with secondary antibody (bs-0295G, 1:20,000). Chemistar ECL Western Blotting Substrate (180-501, Tanon, Shanghai, China) was used to visualize the protein bands. All antibodies were obtained from Bioss (Beijing, China), and the primary antibodies containing E-cadherin (bs-1519R, 1:1000), Vimentin (bs-23063R, 1:1000), α-SMA (bsm-52392R, 1:5000), SMAD4 (bs-23966R, 1:1000), SMAD2 (bs-0718R, 1:1000), p-SMAD2 (bs-20341R, 1:300), SMAD3 (bsm-52224R, 1:1000), p-SMAD3 (bs-5235R, 1:1500), and GAPDH (bs-10900R, 1:2000). All antibodies were diluted with Primary Antibody Dilution Buffer (P0023A, Beyotime). Non-treated and non-transfected cells were used as Control.

### Dual-luciferase reporter assay

The fragments of NEAT1 and SMAD4 3′UTR containing the predicted miR-486-5p binding sites or mutant binding sites were amplified and cloned into pGL3 reporter vector (Promega, Madison, WI, USA), recorded as wild-type and mutant-type NEAT1 or SMAD4 3′UTR reporter vectors (NEAT1-WT/MUT or SMAD4 3′UTR-WT/MUT). SRA01/04 cells were co-transfected with the reporter vectors and miR-486-5p mimic or miR-NC, and the luciferase activities were evaluated using the Dual-Lucy Assay Kit (D0010, Solarbio, Beijing, China).

### RNA immunoprecipitation (RIP) assay

Magna RIP Kit (17-700) was bought from Millipore. Magnetic beads were pre-coated with antibodies against immunoglobulin G (IgG) or argonaute2 (Ago2) overnight at 4 °C. SRA01/04 cells were lysed and then incubated with magnetic beads. After washed with RIP buffer, total RNA was isolated and the abundances of NEAT1, miR-486-5p and SMAD4 were measured by qRT-PCR.

### Biotin-labeled RNA pull-down assay

The biotinylated miR-486-5p (bio-miR-486-5p) probe and negative control (bio-miR-NC) probe were synthesized by Sangon (Shanghai, China) and transfected into SRA01/04 cells. After incubated for 48 h, the cells were harvested and then incubated with Dynabeads M-280 Streptavidin (11205D, Invitrogen). The enrichment of NEAT1 and SMAD4 was determined by qRT-PCR.

### Statistical analysis

Each experiment was carried out at least three times. All statistical analyses were performed using GraphPad Prism 6.0 (GraphPad, La Jolla, CA, USA). Data were represented as mean ± standard deviation. Student’s *t*-test or one-way analysis of variance was used for statistical analysis. The correlation between miR-486-5p and NEAT1 or SMAD4 was determined using Pearson correlation analysis. *P* < 0.05 indicated statistical significance.

## Results

### The expression of NEAT1 and miR-486-5p in PCO patients and TGF-β2-stimulated SRA01/04 cells

**Fig. 1:**
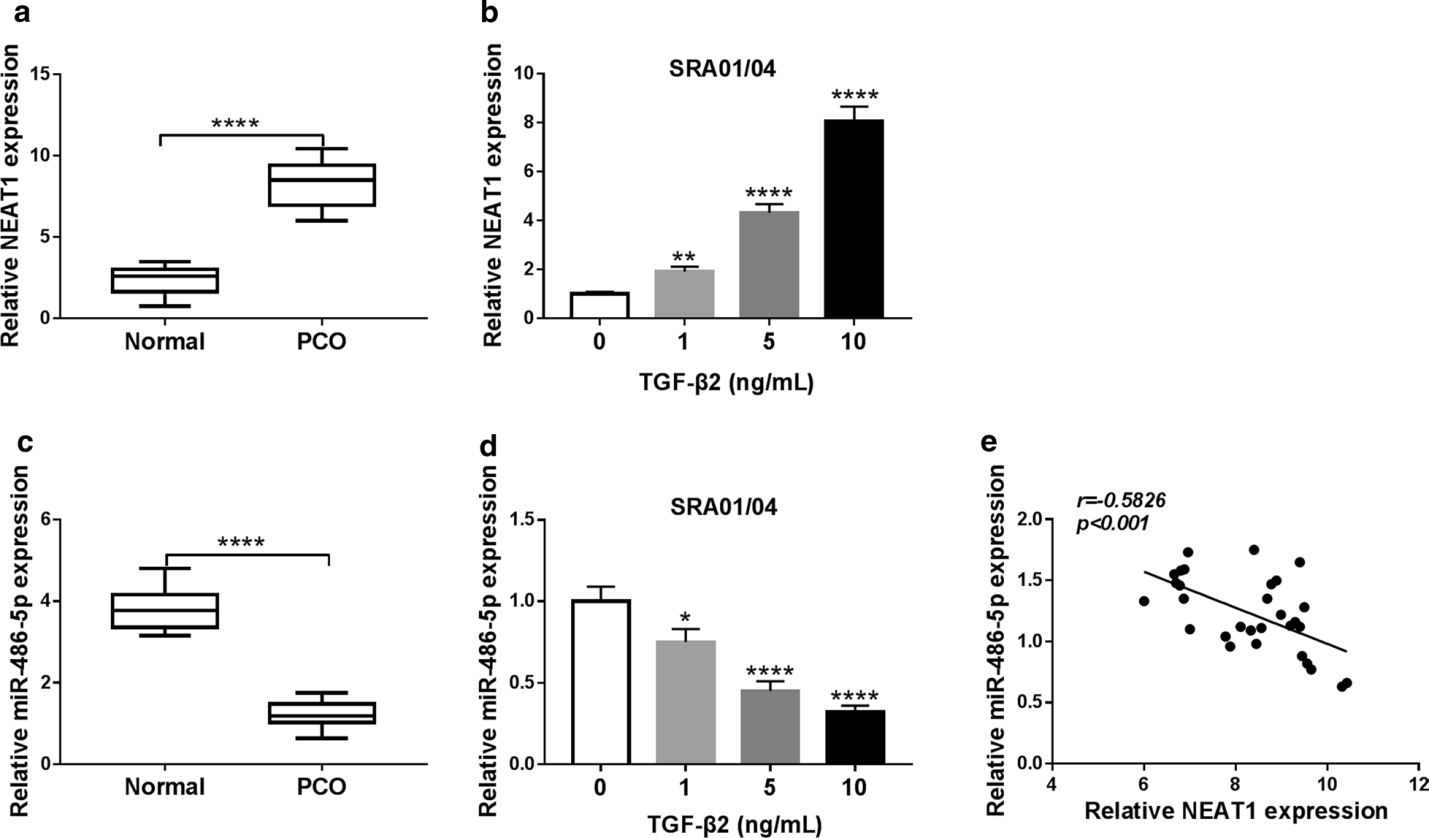
The expression of NEAT1 and miR-486-5p in PCO patients and TGF-β2-stimulated SRA01/04 cells. **a**, **b** QRT-PCR analysis showing the increased levels (*P *< 0.0001) of NEAT1 in posterior capsular tissues from PCO patients and in TGF-β2-treated SRA01/04 cells. **c**, **d** QRT-PCR analysis suggested that the expression of miR-486-5p was decreased (*P *< 0.0001) in posterior capsular tissues from PCO patients and in TGF-β2-treated SRA01/04 cells. **e** Pearson correlation analysis revealed that NEAT1 expression was negatively correlated with miR-486-5p in posterior capsular tissues of PCO patients. *P < 0.05, ****P < 0.0001

To identify the function of NEAT1 in PCO, we first detect its expression in posterior capsular tissues. As shown in Fig. 1a, NEAT1 expression is elevated in the posterior capsular tissues of PCO patients compared to the normal posterior capsular tissues. Moreover, in TGF-β2-stimulated SRA01/04 cells, NEAT1 expression is increased in a dose-dependent manner (Fig. 1b). At the same time, we also measure the miR-486-5p expression and discover that miR-486-5p is lower expressed in PCO patients compared with that in normal humans (Fig. 1c). Besides, miR-486-5p also has decreased expression in TGF-β2-stimulated SRA01/04 cells in a dose-dependent manner (Fig. 1d). Through the correlation analysis, we discover that miR-486-5p expression is negatively correlated with NEAT1 (Fig. 1e). Therefore, we speculate that NEAT1 and miR-486-5p may have important roles in the development of PCO.

### Knockdown of NEAT1 suppresses proliferation, migration, invasion and EMT in TGF-β2-stimulated SRA01/04 cells

**Fig. 2:**
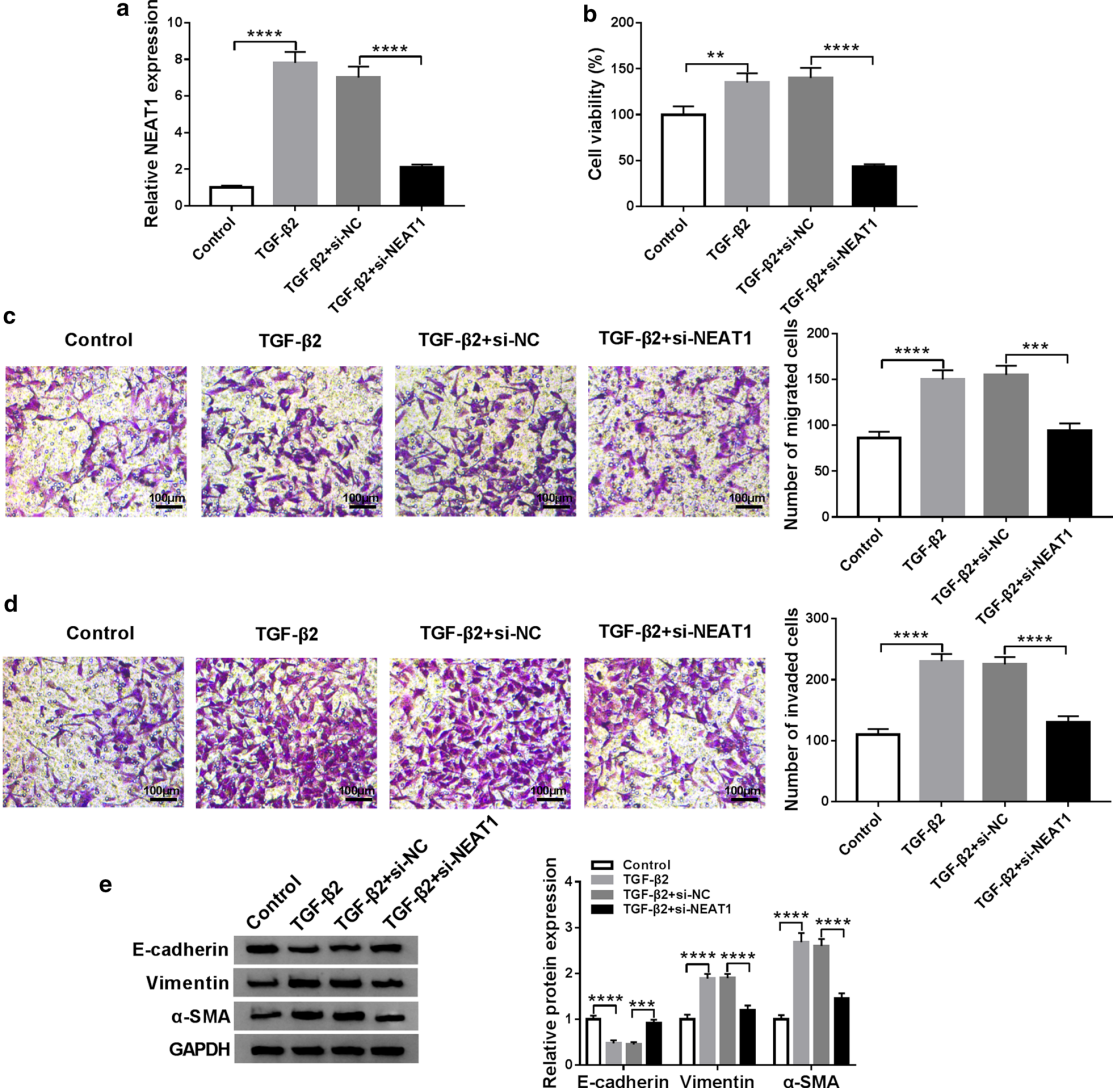
Effect of TGF-β2 and NEAT1 knockdown on the progression of SRA01/04 cells. SRA01/04 cells were transfected with si-NC or si-NEAT1, and then treated with 10 ng/mL TGF-β2. **a** QRT-PCR analysis showed that si-NEAT1 could inhibit NEAT1 expression (*P *< 0.0001) in TGF-β2-treated SRA01/04 cells. **b** MTT assay indicated that NEAT1 silencing reduced the proliferation of TGF-β2-treated SRA01/04 cells (*P *< 0.0001). **c**, **d** Transwell assay revealed that NEAT1 knockdown suppressed the migration and invasion of TGF-β2-treated SRA01/04 cells (100 μM) (*P *< 0.001, *P *< 0.0001). **e** WB analysis showed that silenced NEAT1 enhanced E-cadherin protein level, while inhibited Vimentin and α-SMA protein levels (*P *< 0.001, *P *< 0.0001). **P < 0.01, ***P < 0.001, ****P < 0.0001

Subsequently, we use the si-NEAT1 to explore the effect of NEAT1 knockdown on the proliferation, migration, invasion and EMT of TGF-β2-stimulated SRA01/04 cells. The concentration of TGF-β2 used is 10 ng/mL. The reversing effect of si-NEAT1 on TGF-β2-induced NEAT1 expression confirms its high transfection efficiency (Fig. 2a). Further, MTT assay results suggest that TGF-β2 promotes the proliferation of SRA01/04 cells, while NEAT1 silencing can invert this effect (Fig. 2b). Also, the acceleration effect of TGF-β2 on the numbers of migrated and invaded SRA01/04 cells also can be recovered by NEAT1 knockdown, indicating that silenced NEAT1 represses the migration and invasion of SRA01/04 cells (Fig. 2c, d). Meanwhile, we also determine the protein levels of EMT marker proteins. As presented in Fig. 2e, TGF-β2 hinders the E-cadherin protein level and enhances the Vimentin and α-SMA protein levels, showing that TGF-β2 can induce the EMT process of SRA01/04 cells. However, si-NEAT1 can change the effect of TGF-β2 on the E-cadherin, Vimentin and α-SMA protein expression, thus reversing the promoting effect of TGF-β2 on the EMT process of SRA01/04 cells. Our results suggest that NEAT1 may have an essential function in regulating the proliferation, metastasis and EMT of LECs.

### NEAT1 interacts with miR-486-5p

**Fig. 3:**
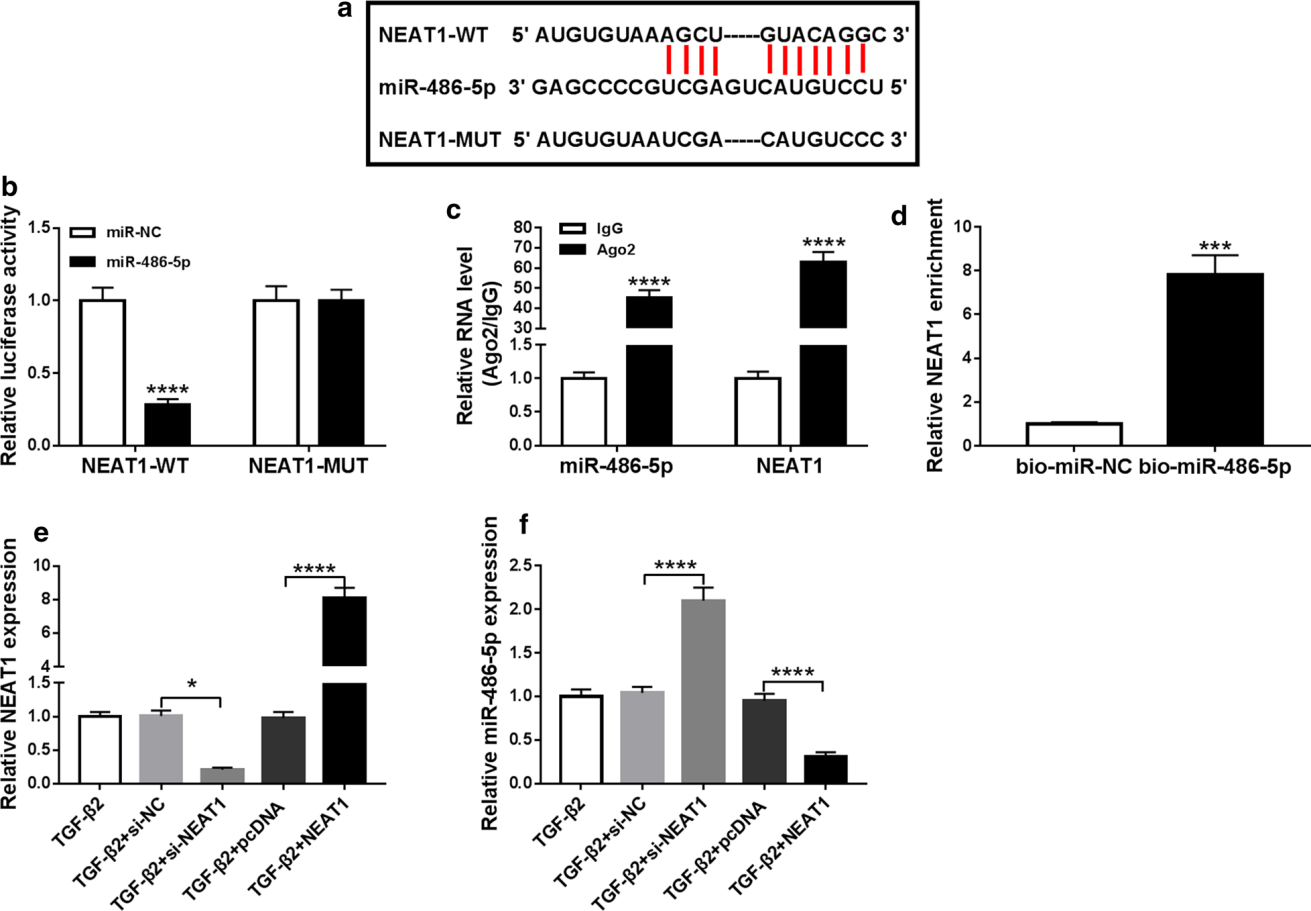
NEAT1 interacted with miR-486-5p. **a** The sequences of NEAT1-WT and NEAT1-MUT were shown. **b** Dual-luciferase reporter assay suggested that the luciferase activity of NEAT1-WT could be inhibited by miR-486-5p (*P *< 0.0001). **c** RIP assay showed that the enrichment of NEAT1 and miR-486-5p was enhanced in Ago2 (*P *< 0.0001). **d** Biotin-labeled RNA pull-down assay suggested that NEAT1 enrichment was increased in the bio-miR-486-5p probe (*P *< 0.001). **e** QRT-PCR analysis indicated that NEAT1 expression was decreased by si-NEAT1 (*P *< 0.05) and promoted by NEAT1 overexpression vector (*P *< 0.0001) in TGF-β2-stimulated SRA01/04 cells. **f** QRT-PCR analysis showed that miR-486-5p expression was increased by NEAT1 knockdown (*P *< 0.0001) and reduced by NEAT1 overexpression (*P *< 0.0001) in TGF-β2-stimulated SRA01/04 cells. *P < 0.05, ***P < 0.001, ****P < 0.0001

Through the StarBase v2.0 tool, we found that miR-486-5p has binding sites with NEAT1 (Fig. 3a). Besides, we also use the dual-luciferase reporter assay, RIP assay and biotin-labeled RNA pull-down assay to verify the interaction between NEAT1 and miR-486-5p. The results reveal that miR-486-5p overexpression inhibits the luciferase activity of NEAT1-WT vector without affecting that of the NEAT1-MUT vector (Fig. 3b). RIP assay results suggest that the enrichment of miR-486-5p and NEAT1 is significantly increased in Ago2 compared to IgG (Fig. 3c), and biotin-labeled RNA pull down assay results show that NEAT1 enrichment is markedly enhanced in the bio-miR-486-5p probe compared to the bio-miR-NC probe (Fig. 3d). The above data illuminate that NEAT1 can interact with miR-486-5p. Next, we investigate the effect of NEAT1 expression on miR-486-5p expression. The expression of NEAT1 is markedly inhibited by si-NEAT1 and remarkably increased by NEAT1 overexpression vector, indicating that the transfection efficiency of both is good (Fig. 3e). By measuring the miR-486-5p expression, we found that silenced NEAT1 can improve miR-486-5p expression, while overexpressed NEAT1 can hinder its expression (Fig. 3f).

### NEAT1 sponges miR-486-5p to regulate LECs progression

**Fig. 4:**
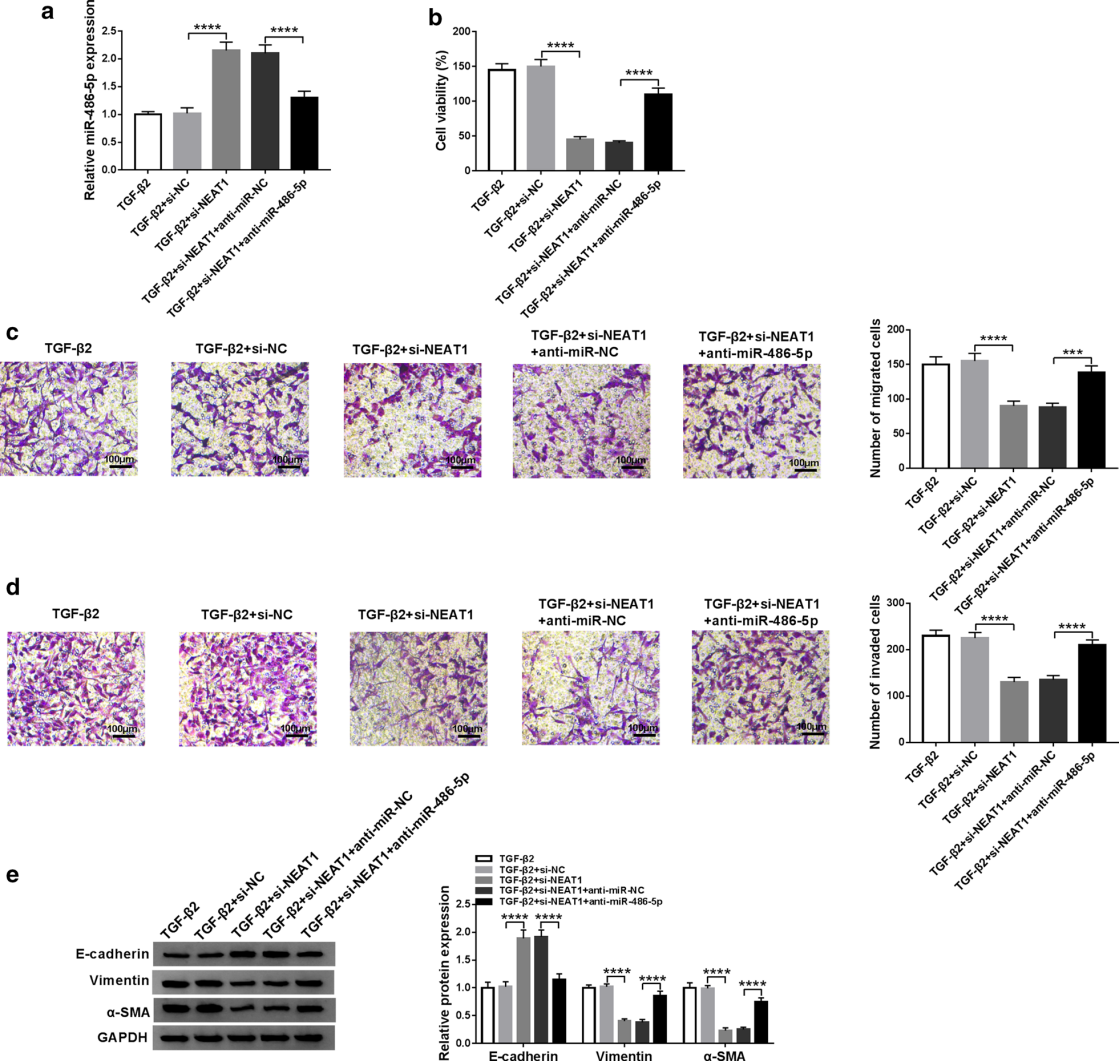
Effects of NEAT1 silencing and miR-486-5p inhibitor on the progression of TGF-β2-stimulated SRA01/04 cells. SRA01/04 cells were co-transfected with si-NEAT1 and anti-miR-486-5p or anti-miR-NC, and then treated with 10 ng/mL TGF-β2. **a** QRT-PCR analysis suggested that miR-486-5p inhibitor reversed the promotion effect of NEAT1 silencing on miR-486-5p expression (*P *< 0.0001). MTT assay (**b**), and transwell assay (**c**, **d**) showed that miR-486-5p inhibitor reversed the inhibition effect of NEAT1 knockdown on the proliferation (*P *< 0.0001), migration (*P *< 0.001) and invasion (*P *< 0.0001) (100 μM) of TGF-β2-stimulated SRA01/04 cells. **e** WB analysis indicated that the regulation of NEAT1 silencing on the protein levels of E-cadherin, Vimentin and α-SMA could be reversed by miR-486-5p inhibitor (*P *< 0.0001) in TGF-β2-stimulated SRA01/04 cells. ***P < 0.001, ****P < 0.0001

To determine whether NEAT1 regulates LECs progression through miR-486-5p, we co-transfect si-NEAT1 and anti-miR-486-5p into TGF-β2-stimulated SRA01/04 cells. Through detecting the expression of miR-486-5p, we confirm that miR-486-5p inhibitor can reverse the promoting effect of NEAT1 knockdown on miR-486-5p expression, suggesting that the transfection of both is effective (Fig. 4a). MTT assay results determine that miR-486-5p inhibitor reverses the inhibitory effect of NEAT1 silencing on the proliferation of TGF-β2-stimulated SRA01/04 cells (Fig. 4b), and transwell assay results also show that the suppression effect of NEAT1 knockdown on the migration and invasion of TGF-β2-stimulated SRA01/04 cells can be inverted by miR-486-5p inhibitor (Fig. 4c, d). In addition, the miR-486-5p inhibitor also reverses the promoting effect of silenced NEAT1 on the E-cadherin expression and the inhibiting effect on the Vimentin and α-SMA expression, thus restoring the EMT process of TGF-β2-stimulated SRA01/04 cells (Fig. 4e). Hence, our results demonstrate that NEAT1 regulates the progression of PCO through miR-486-5p.

### SMAD4 serves as a target of miR-486-5p

**Fig. 5:**
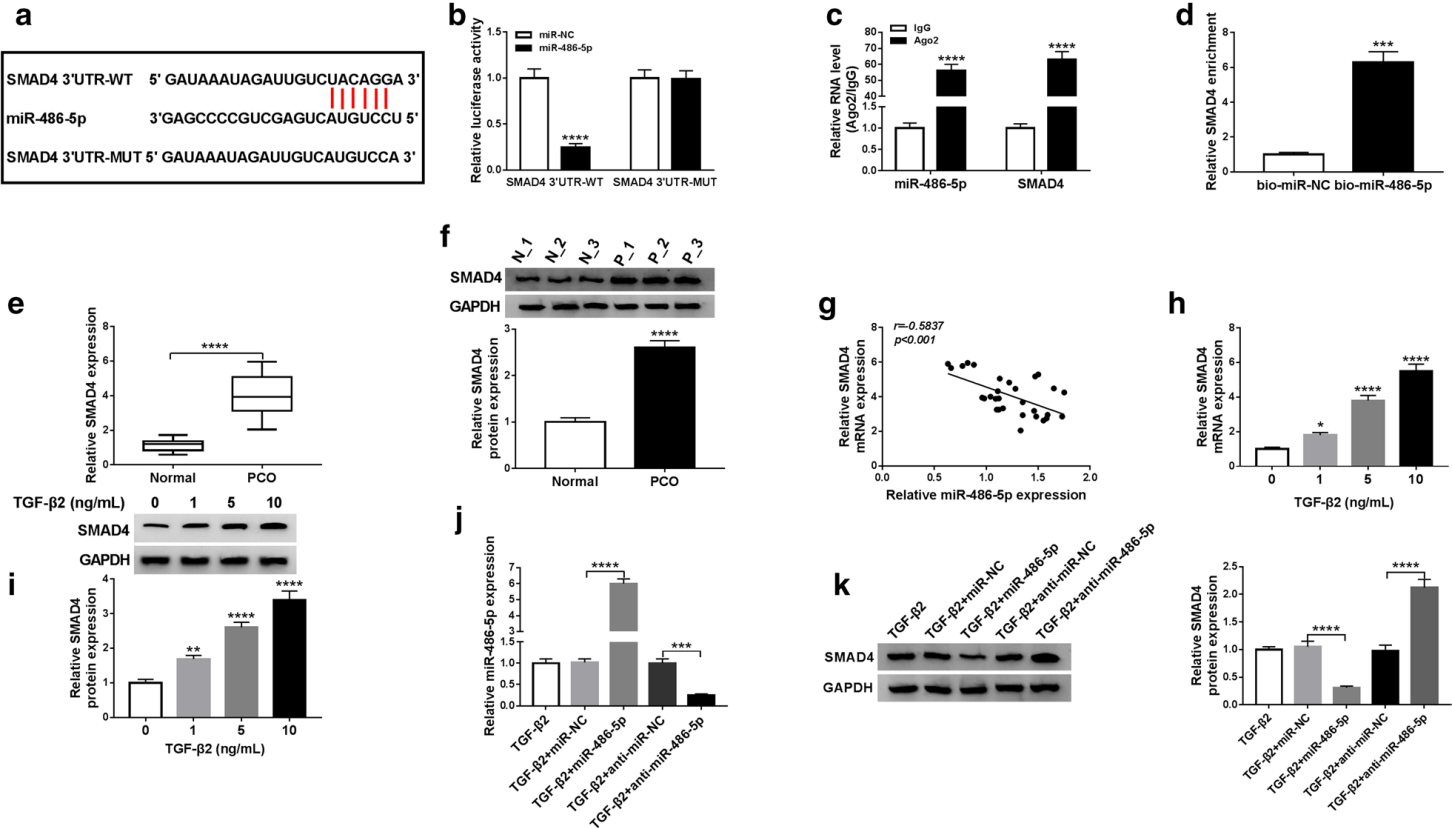
SMAD4 served as a target of miR-486-5p. **a** The fragments of SMAD4 3′UTR-WT and SMAD4 3′UTR-MUT were presented. **b** Dual-luciferase reporter assay indicated that the luciferase activity of SMAD4 3′UTR-WT could be reduced by miR-486-5p (*P *< 0.0001). **c** RIP assay suggested that the enrichment of miR-486-5p and SMAD4 was increased in Ago2 (*P *< 0.0001). **d** Biotin-labeled RNA pull-down assay showed that the enrichment of SMAD4 could be pulled down by the bio-miR-486-5p probe (*P *< 0.001). **e**, **f** QRT-PCR and WB analysis suggested that the mRNA and protein levels of SMAD4 were promoted in posterior capsular tissues from PCO patients compared to normal humans. **g** Pearson correlation analysis revealed that miR-486-5p expression was negatively correlated with and SMAD4 expression in posterior capsular tissues of PCO patients. **h**, **i** QRT-PCR and WB analysis showed that the mRNA and protein levels of SMAD4 were increased in SRA01/04 cells treated with TGF-β2 in a dose-dependent manner (*P *< 0.05, *P *< 0.01, *P *< 0.0001). **j** QRT-PCR analysis revealed that the expression of miR-486-5p was enhanced by miR-486-5p mimic (*P *< 0.0001) while reduced by miR-486-5p inhibitor (*P *< 0.001) in TGF-β2-stimulated SRA01/04 cells. **k** WB analysis presented that the protein level of SMAD4 was decreased by miR-486-5p overexpression (*P *< 0.0001) and increased by measured by miR-486-5p inhibition (*P *< 0.0001). *P < 0.05, **P < 0.01, ***P < 0.001, ****P < 0.0001

For perfecting the mechanism of the NEAT1/miR-486-5p axis, we also use the StarBase v2.0 tool to predict the targets of miR-486-5p. And we discover that SMAD4 has a targeting sequence of miR-486-5p (Fig. 5a). Dual-luciferase reporter assay results indicate that the luciferase activity of SMAD4 3′UTR-WT instead of SMAD4 3′UTR-MUT can be restrained by miR-486-5p overexpression (Fig. 5b). Moreover, RIP assay results show that the levels of miR-486-5p and SMAD4 are enriched in Ago2 compared with IgG (Fig. 5c). Furthermore, we also found that SMAD4 enrichment is remarkably increased in the bio-miR-486-5p probe compared to the bio-miR-NC probe (Fig. 5d). By measuring the mRNA and protein expression of SMAD4, we found that it is highly expressed in the posterior capsular tissues of PCO patients compared with normal posterior capsular tissues (Fig. 5e, f), and negatively correlated with the expression of miR-486-5p (Fig. 5g). Further, SMAD4 is also upregulated in TGF-β2-stimulated SRA01/04 cells with a dose-dependent manner (Fig. 5h, i). Meanwhile, we determine the influence of miR-486-5p expression on SMAD4 expression. The expression of miR-486-5p can be elevated by miR-486-5p mimic and reduced by its inhibitor, confirming the effectiveness of mimic and inhibitor (Fig. 5j). WB analysis results show that miR-486-5p overexpression inhibits the protein level of SMAD4, while miR-486-5p inhibitor increases its protein level (Fig. 5k). These data reveal that miR-486-5p directly targets SMAD4.

### MiR-486-5p regulates the progression of TGF-β2-stimulated LECs through SMAD4

**Fig. 6:**
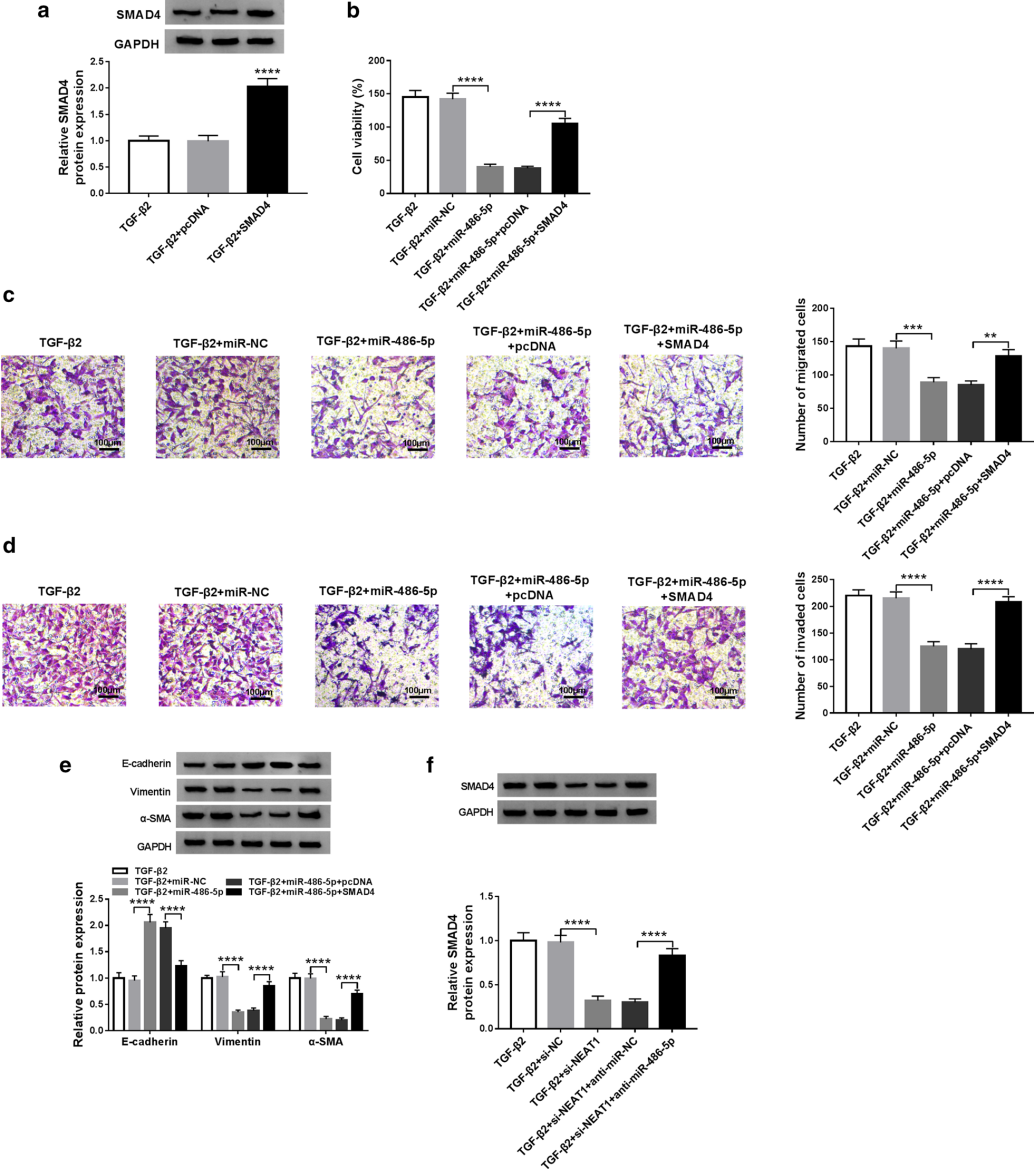
Effects of miR-486-5p overexpression and SMAD4 overexpression on the progression of TGF-β2-stimulated SRA01/04 cells. **a** SRA01/04 cells were transfected with SMAD4 overexpression vector or pcDNA, and then treated with 10 ng/mL TGF-β2. WB analysis showed that the protein level of SMAD4 was promoted by SMAD4 overexpression vector (*P *< 0.0001) in TGF-β2-treated SRA01/04 cells. **b**–**f** SRA01/04 cells were co-transfected with miR-486-5p mimic and SMAD4 overexpression vector or pcDNA, and then treated with 10 ng/mL TGF-β2. MTT assay (**b**) and transwell assay (**c**, **d**) suggested that overexpressed SMAD4 reversed the inhibition effect of miR-486-5p on the proliferation (*P *< 0.0001), migration (*P *< 0.01, *P *< 0.001) and invasion (*P *< 0.0001) (100 μM) of TGF-β2-stimulated SRA01/04 cells. **e** WB analysis revealed that the regulation of miR-486-5p on the protein levels of E-cadherin, Vimentin and α-SMA could be reversed by SMAD4 overexpression (*P *< 0.0001) in TGF-β2-stimulated SRA01/04 cells. **f** WB analysis showed that miR-486-5p inhibitor reversed the decreasing effect of NEAT1 silencing on SMAD4 protein expression (*P *< 0.0001). **P < 0.01, ***P < 0.001, ****P < 0.0001

To confirm the role of SMAD4 in miR-486-5p regulates the progression of LECs, we perform the rescue experiments using miR-486-5p mimic and SMAD4 overexpression vector. The increased protein level of SMAD4 confirm the effectiveness of SMAD4 overexpression vector (Fig. 6a). The results of cell viability show that miR-486-5p has a suppressed effect on the proliferation of TGF-β2-stimulated SRA01/04 cells, whereas SMAD4 overexpression inverts this effect (Fig. 6b). Furthermore, overexpressed SMAD4 also reverses the suppression effect of miR-486-5p overexpression on the migration and invasion of TGF-β2-stimulated SRA01/04 cells (Fig. 6c, d). Similarly, the increasing effect of overexpressed miR-486-5p on the E-cadherin level and the decreasing effect on the Vimentin and α-SMA levels also can be reversed by SMAD4 overexpression (Fig. 6e). In addition, we also found that knockdown of NEAT1 inhibits SMAD4 expression, and this effect can be recovered by the miR-486-5p inhibitor (Fig. 6f). In a word, our results reveal that the regulation effect of the NEAT1/miR-486-5p axis on the progression of LECs is achieved by SMAD4.

### NEAT1 regulates the TGF-β/SMAD signaling pathway by affecting SMAD4 and miR-486-5p in TGF-β2-stimulated SRA01/04 cells

**Fig. 7:**
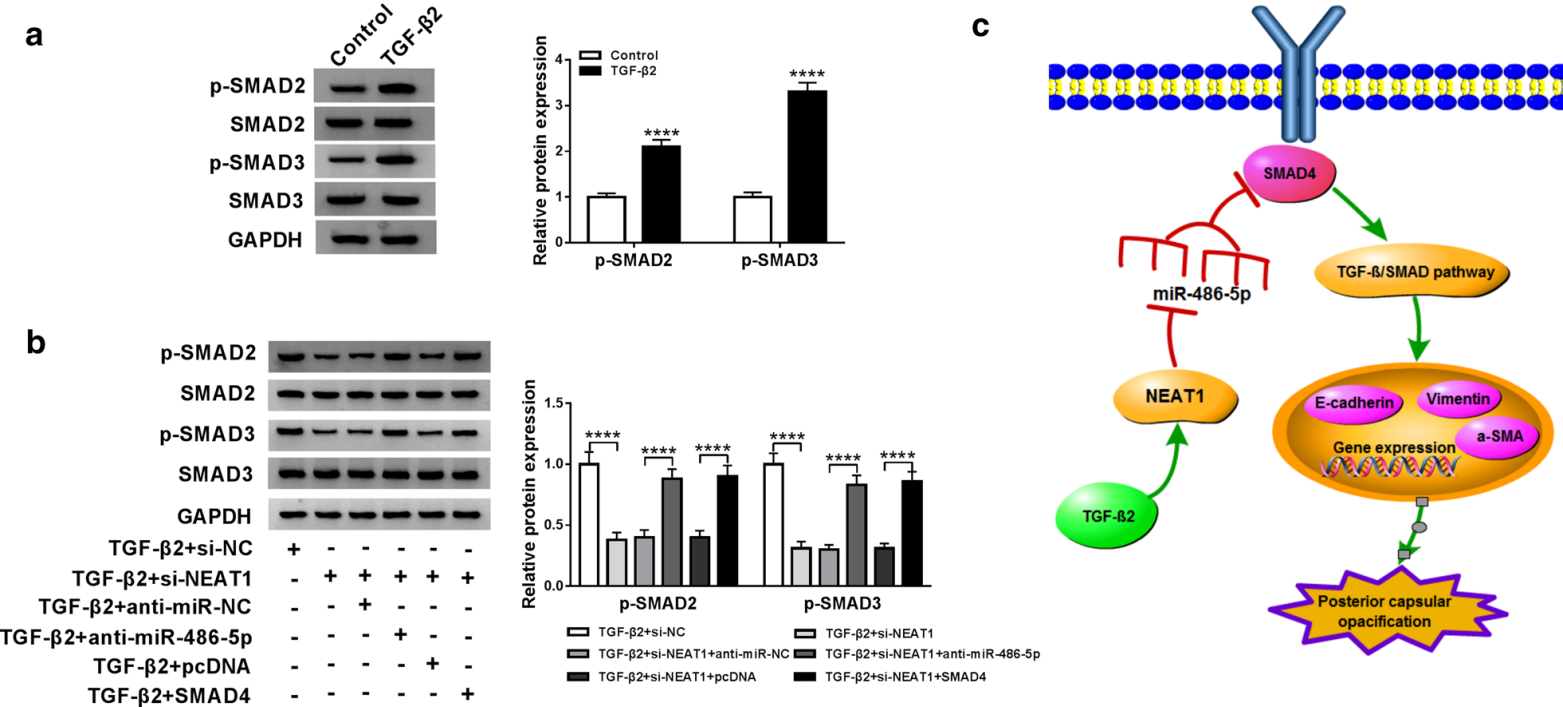
Effects of TGF-β2, NEAT1, miR-486-5p and SMAD4 on the activity of the TGF-β/SMAD signaling pathway. **a** WB analysis suggested that TGF-β2 could promote the protein levels of p-SMAD2 and p-SMAD3 (*P *< 0.0001) in SRA01/04 cells. **b** TGF-β2-stimulated SRA01/04 cells were transfected with si-NEAT1 or co-transfected with si-NEAT1 and anti-miR-486-5p or SMAD4. WB analysis showed that the inhibition effect of NEAT1 silencing on protein levels of p-SMAD2 and p-SMAD3 could be reversed by miR-486-5p inhibitor and SMAD4 overexpression (*P *< 0.0001). **c** The summary diagram of this study was shown. ****P < 0.0001

For determining whether TGF-β/SMAD signaling pathway is involved in the development of NEAT1 regulating the progression of LECs, we detect the expression of p-SMAD2 and p-SMAD3. Through WB analysis, we first confirm that TGF-β2 induces the expression of p-SMAD2 and p-SMAD3 proteins, indicating that TGF-β2 can activate the TGF-β/SMAD signaling pathway (Fig. 7a). Also, we found that interfering of NEAT1 inhibits the expression of p-SMAD2 and p-SMAD3 in TGF-β2-stimulated SRA01/04 cells, and this inhibition is partially restored after the addition of miR-486-5p inhibitor and SMAD4 overexpression vector in TGF-β2-stimulated SRA01/04 cells (Fig. 7b). Therefore, our study confirm that the NEAT1/miR-486-5p/SMAD4 axis mediates the development of LECs by regulating the activity of the TGF-β/SMAD signaling pathway (Fig. 7c).

## Discussion

PCO often leads to progressive loss of vision in patients and can cause vision loss in severe cases, so it brings a lot of inconvenience to the life of patients [27]. At present, Nd-YAG laser capsulotomy is often used for the treatment of PCO, but postoperative complications are still possible [28]. Therefore, a better understanding of the factors affecting the pathogenesis of PCO is conducive to the development of new strategies to prevent and alleviate the development of PCO. NEAT1 is often considered an oncogene in cancer because of its role in promoting proliferation, metastasis, and EMT [29, 30]. Dong et al. found that NEAT1 and MALAT1 were highly expressed in PCO, and confirmed that MALAT1 could promote EMT of TGF-β2-induced LECs by regulating the miR-26a/SMAD4 axis [18]. However, the role of NEAT1 in TGF-β2-induced LECs is unclear. Similarly with the previous study, our study found that NEAT1 was highly expressed in posterior capsular tissues of PCO patients and TGF-β2-stimulated LECs. But not only the EMT of cells, our research also explored the effect of lncRNA on cell proliferation and metastasis. The inhibitory effect of NEAT1 on the proliferation, metastasis and EMT of TGF-β2-stimulated LECs indicated that NEAT1 was a key factor for LECs to maintain normal biological function. Consistent with previous findings [18], our results provide new evidence for NEAT1 as a target for the treatment of PCO.

The involvement of miR-486-5p in disease progression in the form of low expression had been well documented. For example, miR-486-5p was believed to interact with lncRNA DLGAP1-AS1 to participate in the regulation of hepatocellular carcinoma cell proliferation by DLGAP1-AS1 [31]. Also, miR-486-5p had been reported to modulate the EMT process in papillary thyroid cancer by regulating KIAA1199 expression [32]. Therefore, the negative influence of miR-486-5p on cell proliferation, metastasis and EMT has been widely confirmed. In our study, we found that the expression trend of miR-486-5p in posterior capsular tissues of PCO patients and TGF-β2-stimulated LECs was opposite to NEAT1, and verified the interaction between the two through bioinformatics. At the same time, the reversing effect of anti-miR-486-5p on the function of si-NEAT1 also confirmed the negative regulatory effect of miR-486-5p on the proliferation, migration, invasion and EMT of TGF-β2-stimulated LECs. The anti-proliferation, anti-metastasis and anti-EMT effects of miR-486-5p on LECs also were verified by our data, which was consistent with the results of Liu et al. [23]. Hence, miR-486-5p might also be an effective molecular target to prevent PCO progression.

SMAD has been a focus of research because it is a key protein in the TGF-β/SMAD signaling pathway that mediates cell growth and differentiation [25]. Some studies have shown that the low expression of SMAD4 is believed to inhibit cell proliferation, metastasis and EMT, such as in colon cancer and esophageal squamous cell carcinoma [33, 34]. Herein, we suggested that SMAD4 had an increased expression in posterior capsular tissues of PCO patients and TGF-β2-stimulated LECs. The inverting effect of SMAD4 on the function of miR-486-5p mimic confirmed that SMAD4 was the target of miR-486-5p. More importantly, the pro-proliferation, pro-metastasis and pro-EMT effects of SMAD4 on LECs also was demonstrated by this study, which was consistent with previous research [18, 26]. In addition, we also indicated the regulatory effect of the NEAT1/miR-486-5p/SMAD4 axis on p-SMAD2 and p-SMAD3 expression, which confirmed that TGF-β/SMAD signaling pathway was the downstream pathway of the NEAT1/miR-486-5p/SMAD4 axis. These results provided a perfect molecular mechanism for NEAT1 to regulate the progress of PCO.

Of course, there are still some deficiencies in our current research. In the rescue experiment, we found that the reversal effect of miR-486-5p inhibitor on NEAT1 silencing function is partial, so this indicates that there may be other miRNAs involved in the regulation of NEAT1 on the biological functions of LECs. Similarly, the reversal effect of SMAD4 on miR-486-5p function is also partial, which indicates that there may be other targets involved in the regulation of miR-486-5p on the biological functions of LECs. In future research, we will focus on exploring more mechanisms by which NEAT1 regulates the biological functions of LECs, in order to provide new ideas for the treatment of PCO.

## Conclusion

Taken together, the current study showed that NEAT1 might have a promoting effect on the development of PCO. Our results concluded that NEAT1 regulated the proliferation, migration, invasion and EMT of TGF-β2-induced LECs through the miR-486-5p/SMAD4 axis. These findings might provide a new target and theoretical basis for molecular targeted therapy of PCO, and had great clinical significance.

## Data Availability

The analyzed data sets generated during the present study are available from the corresponding author on reasonable request.

## Abbreviations

LECs: Lens epithelial cells
EMT: Epithelial-mesenchymal transformation
PCO: Posterior capsular opacification
SMAD4: *Drosophila* mothers against decapentaplegic 4
qRT-PCR: quantitative real-time polymerase chain reaction
NEAT1: Nuclear enriched abundant transcript 1
DMEM: Dulbecco’s modified Eagle’s medium
WB: Western blot
